# Evaluating the role of quality assessment of primary studies in systematic reviews of cancer practice guidelines

**DOI:** 10.1186/1471-2288-5-8

**Published:** 2005-02-16

**Authors:** Melissa C Brouwers, Mary E Johnston, Manya L Charette, Steve E Hanna, Alejandro R Jadad, George P Browman

**Affiliations:** 1 Department of Clinical Epidemiology and Biostatistics, McMaster University, Hamilton, Canada; 2 Program in Evidence-based Care, Cancer Care Ontario, Hamilton, Canada; 3 University of Toronto and University Health Network, Toronto, Canada; 4 Hamilton Regional Cancer Centre, Hamilton, Canada

## Abstract

**Background:**

The purpose of this study was to evaluate the role of study quality assessment of primary studies in cancer practice guidelines.

**Methods:**

Reliable and valid study quality assessment scales were sought and applied to published reports of trials included in systematic reviews of cancer guidelines. Sensitivity analyses were performed to evaluate the relationship between quality scores and pooled odds ratios (OR) for mortality and need for blood transfusion.

**Results:**

Results found that that whether trials were classified as high or low quality depended on the scale used to assess them. Although the results of the sensitivity analyses found some variation in the ORs observed, the confidence intervals (CIs) of the pooled effects from each of the analyses of high quality trials overlapped with the CI of the pooled odds of all trials. Quality score was not predictive of pooled ORs studied here.

**Conclusions:**

Had sensitivity analyses based on study quality been conducted prospectively, it is highly unlikely that different conclusions would have been found or that different clinical recommendations would have emerged in the guidelines.

## Background

Quality assessment of trials included in systematic reviews of evidence is a resource intensive and scientifically controversial endeavour. On the one hand, the routine use of quality assessment in the development of systematic reviews is encouraged by the Evidence-based Practice Center Program of the Agency for Healthcare Research & Quality (AHRQ) and the Cochrane Collaboration, two well respected groups that coordinate a substantial number of systematic reviews [1-3]. Indeed, West et al released a 2002 evidence report sponsored by the AHRQ comparing and contrasting various systems to rate the strength and quality of research evidence to assist in these activities [4]. Furthermore, many journal editors consider it important to include an assessment of study quality in reports featuring meta-analyses [3].

The concept of incorporating study quality assessment into systematic review methodology has also found empirical support. There is evidence that studies of lower methodological quality tend to report larger treatment effects than high quality studies [5-7]. For example, Moher and his colleagues found a 34% greater estimate of treatment effect for low quality versus high quality trials and a 37% greater estimate of treatment effect for inadequately concealed versus adequately concealed trials associated with reviews addressing a variety of clinical conditions [5]. Similar bias was found by Schultz and his colleagues [6] in their analysis of trials included in the Cochrane Collaboration's Childbirth and Pregnancy reviews. In addition, Colditz and colleagues found that nonrandomized and open studies were more likely to produce positive treatment effects than randomized and double-blinded studies [7].

Although this seminal work yields compelling results, these findings are not universal and the issue is not without detractors [8-14]. Some studies have found no reliable relationship between quality score and effect size [10-12] and another has found that low study quality was associated with diminished effect sizes [13]. Further, Juni et al [14] found the relationship between study quality and effect size depended on the scale used in the assessment.

Together these results suggest the study quality issue is controversial and that the merits of this methodological step in systematic review requires thoughtful analysis. Indeed, West et al conclude with recommendations advocating for research dedicated to comparing quality rating systems and the role of quality assessment within individual clinical contexts and for studies targeted at determining specific quality factors that make a difference in final quality scores [4].

The Practice Guidelines Initiative of the Cancer Care Ontario's Program in Evidence-based Care (PEBC) uses the Guidelines Development Cycle to create cancer practice guidelines comprised of a systematic review of the research literature, an interpretation and consensus of the evidence by members of the guideline development team, clinical recommendations informed by the evidence, and an external review process by Ontario clinicians [15-19]. We face the challenge of balancing scientific rigour and the timely production of guideline documents in an environment defined by limited financial and human resources. Hence, we try to approach our methodological decisions with a critical scientific and practical eye. We took note of the growing controversy in the study quality assessment literature and conducted an evaluation, reported below, to evaluate the benefits of assessing the quality of each study included in our systematic reviews. Our overall objective was to decide whether to augment our current practice of simply describing study characteristics to also incorporate study quality assessment as a routine formal component of the guideline development methodology. The evaluation was conducted in three steps, each of which was designed to address three specific issues:

1. What valid and reliable quality assessment instrument would be most appropriate for our context?

2. How is study quality currently being used in published systematic reviews of cancer trials and what is the relationship between effect size and study quality in this disease area?

3. What impact would study quality assessment have on the clinical recommendations made in evidence-based practice guidelines developed by the PEBC?

## Methods

### Search for a valid and reliable quality assessment tool for the PEBC context

For a comprehensive review of the strengths and weaknesses of quality assessment instruments, readers are referred to the 2002 West et al. evidence report commissioned by the AHRQ [4]. For our study, which began before the release of this report, we used components of Moher et al's definition of study quality that are related to internal validity (i.e., design, conduct and analysis) [20] and updated his 1992 published reports of check lists and scales used to measure the phenomenon [21]. We searched the Medline database using the following search strategy: (quality adj rat:).tw OR (quality adj assess:).tw. OR (quality adj scale:).tw. OR (quality adj checklist:).tw. AND randomized controlled trials.sh. OR clinical trial:.tw. OR random:.tw. Reference lists of reviews were scanned for additional citations.

### Systematic review of the oncology literature on study quality

To locate systematic reviews on oncology topics, the strategy suggested by Moher et al for finding systematic reviews [3] was combined with the terms "neoplasms.sh. OR cancer.tw. OR carcinoma.tw" to search the Medline, CINAHL, and Cancerlit databases. To ascertain if the authors assessed the quality of the studies included in the systematic reviews, the search was narrowed to include the terms [(quality adj rat:).tw OR (quality adj assess:).tw. OR (quality adj scale:).tw. OR (quality adj checklist:).tw. OR (study adj quality).tw.]. Textwords were used to search the Cochrane Library for systematic reviews on oncology topics. Systematic reviews that included analyses exploring the relationship between study quality (using any assessment instrument, not just validated tools that met our criteria as described above) and effect size were examined. Because survival following cancer treatment is commonly used as the primary outcome variable in our practice guidelines, this variable was selected as the primary outcome measure of interest.

### Impact of study quality on PEBC practice guidelines

The validated scales were applied by two methodologists (MJ and MC) to studies reported in any our practice guidelines that included a pooled analysis based on at least ten randomized trials related to the main guideline question. Intraclass correlation coefficients with 95% confidence intervals (CI) were calculated using a random sample of the RCTs to assess inter-rater reliability, one coefficient calculated for each of the scales used. Because of budgetary limitations for staff time, the analysis was conducted on 18 randomly selected studies rather on the whole group of articles. This is a methodological limitation as fewer studies result in larger confidence intervals and less precise estimates. This may account for the difference in reliability ratings we found for the Sindu scale compared to published norms (see Table 1).

**Table 1:** Quality assessment tools with published validity and reliability data – information reported by the scale developers

	**Instrument Description**	**Item Generation**	**Validity**	**Inter-Rater Reliability**	**Application in Study**
**Jadad et al (22)**	*number of items: *3 or 6 *scoring for 3 item: *0, 1, or 2 for two items 0 or 1 for 1 item range 0 to 5 *scoring for 6 item: *0, 1, or 2 for two items 0 or 1 for 4 items range 0 to 8 *cut offs *not specified for either version	consensus among 6 judges (pain management &/or instrument development) pretested with 3 raters on 13 clinical trial reports	3 groups of pain studies were identified a priori: • studies rated as excellent by experts (n = 7); overall score = 3.4 • studies rated as poor by experts (n = 6); overall score = 0.7 • a random selection of RCTs from a MEDLINE search (n = 23); overall score = 2.7	ICC = 0.66 (95% CI: 0.53, 0.79) 14 raters	3-item and 6-item versions in their entirety

**Sindhu et al (24)**	*number of items: *53 *scoring: *items weighted from 1 to 10 range 0 – 100 *cut offs *not specified	8-member Delphi panel (clinicians and methodologists)	Compared with the Chalmers scale for five studies of non-pharmacologic nursing interventions for pain management; r = 0.94 (Pearson) 11 studies considered for a published meta-analysis on hypertension were evaluated: • 9 included studies scored 73–93%; • 2 rejected studies scored below 70%	ICC = 0.93 2 raters	12 of 53 items eliminated because of difficulty applying to cancer studies 41 items related to 12 domains range 0 – 79.5

**Downs & Black (25)**	*number of items: *27 *scoring *0 or 1 for 25 items 1 or 2 for 1 item 0 to 5 for 1 item *cut offs *not specified	based on epidemiological principles, reviews of study designs and existing checklists pilot tested by 2 epidemiologists rating 20 studies	Compared with the Standards of Reporting Trials Group checklist scale for 10 RCTs on surgery for stress incontinence; r = 0.90 (Spearman)	Spearman r = 0.75 2 raters	used 13 internal validity items only items not related to our definition of quality eliminated (10 reporting items, 3 external validity items, 1 power item) range 1 to 13

RCT, randomized controlled trial; ICC, intraclass correlation coefficient; CI, confidence interval

To assess the impact of study quality on effect sizes, sensitivity analyses were conducted for the meta-analysis from each guideline report. For each scale, studies were divided into two groups (low quality and high quality) based on total quality score. Where the scale developer suggested a cut-off point for low versus high quality, this was used. Where no cut point was specified, the observed median study quality score was used as the dividing point between low and high quality. Meta-analyses were repeated with the high quality studies. Because there would never be a situation in which guideline developers would consider low quality studies only, a meta-analysis using this sample of the studies was not conducted.

## Results

### Valid and reliable quality assessment tools for our context

Four scales meeting our criteria were found; two instruments, Jadad et al [22] and Cho & Bero [23], were originally uncovered in the Moher review [21] and two instruments, Sindhu et al [24] and Downs & Black [25], were uncovered in our update of this review. While none of these scales were developed in the oncology setting, they all purport to be generic assessment tools that measure the quality of specific study designs regardless of clinical condition reflected in the design. The procedures undertaken to create the instruments followed appropriate methodological processes for questionnaire design. In addition, while a number of additional scales and checklists emerged from our search, validity and reliability data were not reported. Because our practice guidelines are based primarily on evidence from randomized trials, we decided to reserve to employ the scales that focused specifically on RCTs. As such, the Cho & Bero scale, which is applicable to a range of study designs, was not employed here but will be considered at a later date when we have a portfolio of diverse study designs. The characteristics of the instruments included in our study are summarized in Table 1 and 2 and detailed descriptions and comparisons can be found in West et al. [4].

**Table 2:** Quality assessment tools – comparison of key quality constructs

	**Jadad**	**Sindhu**	**Downs & Black**
Randomization	Max.= 2 points out of total score of 5 1. Was the study described as randomized? (1 point for yes) Give an additional point if the method to generate the sequence of randomization was described and it was appropriate. Deduct a point if it was inappropriate.	Max. = 10 points out of total score of 100 2. Have the patients been randomly allocated to treatment groups? (1 point for yes) If yes: i) Is the method of randomization explicitly detailed? (1.5 points) ii) Is it valid? i.e. Are there any threats to internal validity re: designation of subjects to groups? (2.5 points) iii) Is patient consent sought prior to randomization? (2.5 points) iv) Is it secure and 'blind' to the assessors? (2.5 points)	Max. = 2 points out of total score of 13 23. Were the study subjects randomised to intervention groups? Studies which state that subjects were randomised should be answered yes except where method of randomisation would not ensure random allocation. For example alternate allocation would score no... (1 point for yes) 24. Was the randomised intervention assignment concealed from both patients and health care staff until recruitment was complete and irrevocable? (1 point)

Blinding	Max. = 2 points out of total score of 5 2. Was the study described as double blind? (1 point) Give an additional point if the method of double blinding was described and it was appropriate. Deduct a point if it was inappropriate.	Max. = 5 points out of total score of 100 9. Is the assessment blind? a) If yes, who is blinded: i) patients? (2 points) ii) therapist/carer? (2 points) iii)assessor/data collector? (1 point) b) If no, i) are reasons given as to why assessment is not blind? (2 points) ii) Is there discussion of bias resulting from non-blind assessment? (3 points)	Max. = 2 points out of total score of 13 14. Was an attempt made to blind study subjects to the intervention they have received? (1 point) 15. Was an attempt made to blind those measuring the main outcomes of the intervention? (1 point)

Withdrawals and dropouts Intention to treat analyasis	Max.= 1 point out of total score of 5 3. Was there a description of withdrawals and dropouts? 1 point)	Max. = 12 points out of total score of 100 6. Has an 'intention-to-treat analysis been performed? i.e. everyone randomized is retained in the study; everyone randomized is included in the final analysis; and no selective dropouts. (8 points) b) if not, is it clear what was done, its justification and impact on bias? (8 points) 11. Loss to follow-up a) (<) 20% loss to follow up (2 points) b) <10% loss to follow-up (2 points)	Max. = 2 points out of total score of 13 25. Was there adequate adjustment for confounding in the analyses from which the main findings were drawn? The questions should be answered no for trials if: the main conclusions of the study were based on analyses of treatment rather than intention to treat;.... (1 point) 26. Were losses of patients to follow-up taken into account? If the number of patients lost to follow-up are not reported, the question should be answered unable to determine. If the proportion lost to follow-up was too small to affect the main findings, the question should be answered yes? (1 point)

Appropriate statistical analysis	no items	Max. = 6 points out of total score of 100 7. Statistical analysis a) Is the analysis appropriate/specific to the hypothesis and to the data? (1 point) b) Is the analysis adequately described? (1 point) c) Do the statistical assumptions hold? (1 point) d) Are adequate summary statistics provided at: i) baseline? (0.5 point) ii) outcome? (05. point) e) Is the overall significance level reported protected against inflation due to multiple testing? (1 point) f) If confounders exist, are they adjusted for via multivariate techniques even if differences between groups are not significant? (1 point)	Max. = 4 points out of total score of 13 16. If an of the results of the study were based in 'data dredging', was this made clear? (1 point) 17. Do the analyses adjust for different lengths of follow-up of patients? (1 point) 18. Were the statistical tests used to assess the main outcomes appropriate? (1 point) 25. Was there adequate adjustment for confounding in the analyses from which the main findings were drawn? (1 point)

Compliance with treatment	no items	Max. = 4 points out of total score of 100 14. Has patient compliance been assessed? (4 points)	Max. = 1 point out of total score of 13 19. Was compliance with the interventions reliable? (1 point)

Outcome Measures	no items	Max. = 14 points out of total score of 100 3. Measurement of outcomes a) Is the form of measurement stated? (3 points) b) Has an attempt been made to validate the measures? (3 points) c) Has an attempt been made to test the reliability of the measures? (2 points) d) Is the outcome objective as compared to subjective? (2 points) 12. Outcomes a) How many outcomes are used (1/2 point for each, to a max. of 2) b) Are they relevant? (1 point) c) Are they independent? (1 point)	Max. = 1 point out of total score of 13 20. Were the main outcome measures used accurate (valid and reliable)? (1 point)

### The relationship between study quality and effect size in the oncology literature

The literature review located 32 published systematic reviews on oncology-related topics that included some measure of study quality. Five of the reviews examined changes in pooled estimates of effect size of mortality rates when meta-analysis was restricted to high-quality randomized trials [26-30]. As shown in Table 3, four of the five reviews found somewhat larger effects (i.e., larger differences between experimental and control groups) with high-quality trials compared to all trials [26-29]. With one exception, the statistical relevance of the differences between the groups (i.e., significant differences or no significant differences) remained the same regardless of the number of trials included. Specifically, two of the reviews did not detect a statistically significant difference in survival between groups when all studies were included or when the meta-analysis was restricted to high-quality studies [26,29]. For one data set, the meta-analysis was repeated with study quality ratings used as weights [29]; there was still no significant difference between experimental and control groups. Two analyses detected significant differences between experimental and control treatments with analysis of all trials and when the analysis was restricted to high-quality trials [27,28]. In the fifth review, a significant difference between experimental and control interventions was detected when all trials were synthesized that became only marginally significant (p < .07) when the meta-analysis was adjusted for study quality [30].

**Table 3:** Systematic reviews of randomized oncology trials with sensitivity analysis exploring the relationship between study quality scores and effect sizes for mortality

**Systematic Review**	**Interventions**	**Outcome**	**Quality Scale**	**Definition of High Quality**	**Effect Size (95% confidence interval)**	
					
					**All Studies (# studies)**	**High Quality (# studies)**
McAlister et al, 1998 (26)	allogenic blood transfusion versus autologous or leucocyte-depleted allogenic blood during cancer surgery	relative risk of death*	Jadad (22)	score ≥3 out of 5	RR, 0.94 (0.76 to 1.16) (n = 5)	RR, 0.84 (0.47 to 1.52) (n = 2)
Caubet et al, 1997 (27)	nonsteroidal anti-androgens (plus LHRH or orchiectomy) versus LHRH or orchiectomy alone for advanced prostate cancer	relative risk of death*	Chalmers (31)	score ≥50 % of total possible score	RR, 0.81 (0.70 to 0.94) (n = 13)	RR, 0.78 (0.66 to 0.92) (n = 4)
Dube et al, 1997 (28)	adjuvant chemotherapy versus control for colorectal cancer	odds ratio for death*	Chalmers (31)	score >50 % of total possible score	OR, 0.82 (0.77 to 0.89) (n = 29)	OR, 0.77 (0.71 to 0.85) (n = 14)
Detsky et al, 1992 (29)	total parenteral nutrition versus control in cancer patients undergoing chemotherapy	odds ratio for survival**	Chalmers (31)	score >42 % of total possible score; quality score also used as a weighting factor in meta analysis	OR, 0.74 (0.42 to 1.3) (n = 8)	OR, 0.69 (0.38 to 1.3) (n = 2) weighted OR, 0.61 (0.23 to 1.6)
Klein et al, 1986 (30)	total parenteral nutrition versus control in cancer patients undergoing surgery	odds ratio for operative death*	Developed specifically for the systematic review	quality score used as a weighting factor in meta analysis	OR, 0.44 (0.21 to 0.90, p = 0.02) (n = 10)	weighted OR not reported but p = 0.07 after weighting for study quality

LHRH, luteinizing hormone-releasing hormone; RR, relative risk; OR, odds ratio

*RR or OR <1.0 indicates fewer deaths in the experimental group than in the control group

** OR <1.0 indicates more deaths in the experimental group than in the control group

### Impact of study quality on PEBC practice guidelines

Three of the PEBC practice guidelines included at least 10 RCTs in their systematic reviews of the evidence and were eligible for inclusion in this evaluation [31-33]: concomitant chemotherapy and radiotherapy in squamous cell head and neck cancer (18 trials) [31]; adjuvant therapy for stage II colon cancer following complete resection (11 trials) [32]; and neoadjuvant chemotherapy in locally advanced squamous cell carcinoma of the head and neck (23 trials) [33]. For the latter guideline [33], data could not be reliably reconstructed and is not discussed further.

At the conclusion of our study, we identified a fourth practice guideline which originally did not meet our 10 RCT inclusion criteria, but later did so after it was updated. The guideline focused on the role of erythropoietin (EPO) in the management of cancer patients with non-hematologic malignancies [34]. Unlike the chemotherapy trials included in the practice guidelines described above, which were not placebo-controlled and where the primary outcome was death, one-third of the EPO trials were double blind and all used the need for blood transfusion as the primary outcome. Although by the time this practice guideline emerged as eligible we had identified a preferred scale (see below), we chose to include it here and apply only the preferred scale as a demonstration of its use on a report that had differing characteristics than the chemotherapy topics covered.

#### Inter-rater reliability

Intraclass correlation coefficients used to established inter-rater reliability were 0.71 for the 3-item Jadad scale (95% CI, 0.38 to 0.88), 0.80 for the 6-item Jadad scale (95% CI, 0.54 to 0.92), 0.62 for the Sindhu scale (95% CI, 0.24 to 0.84), and 0.63 for the Downs & Black Scale (95% CI, 0.25 to 0.84). Disagreements were resolved by consensus. Where consensus could not be reached, a third rater (MB) assessed the items and provided the tiebreaker score.

#### Application of quality scales to primary studies informing practice guidelines

While the total quality scores emerging from each of the different scales did all significantly correlate with one another (range r = .35 to r = .73), there was considerable variation in the classification of studies as high quality or low quality as a function of the scale that was applied (Table 4). For example, of the 11 comparisons from 11 trials comprising the stage II colon cancer review, the application of the Jadad 3-item, the Jadad 6-item, the Sindhu, and Downs & Black scales yielded 0, 8, 9, and 6 of these as high quality, respectively. The 6 studies categorized as high quality using the Downs & Black tool were also categorized as high quality when the Jadad 6-item and Sindhu scales were applied. Similarly, the 8 studies categorized as high by the Jadad 6-item were also categorized as high quality by the Sindhu scale.

**Table 4:** Meta-analysis of all trials and high-quality trials from evidence-based practice guidelines

			**Practice Guideline**		
			*Colon Cancer *Adjuvant chemotherapy *Outcome: *Mortality (32)	*Head & Neck *Concomitant therapy *Outcome: *Mortality (31)	*Systemic Therapy *erythropoietin *Outcome: *need for blood transfusion (34)

**All Studies**		# studies (# comparisons)	11 (11)	18 (20)	15 (15)
		OR (CI)	0.82 (0.62 – 1.09)	0.62 (0.54 – 0.72)	0.57 (0.47–0.70)
**High Quality Studies By Assessment Scale Criteria**	Jadad (3-item) range = 0–5 criteria >2	# studies (# comparisons)	0	2 (2)	not applied
		OR (CI)	-	0.54 (0.34 – 0.86)	
	Jadad (6-item) range = 0–8 criteria >3	# studies (# comparisons)	8 (8)	13 (14)	9 (9)
		OR (CI)	0.88 (0.69–1.13)	0.53 (0.44 – 0.64)	0.57 (0.44–0.72)
	Sindhu (41-item) range = 0–79.5 criteria > 44.8	# studies (# comparisons)	9 (9)	11 (12)	not applied
		OR (CI)	0.86 (0.68–1.09)	0.62 (0.49 – 0.79)	
	Downs & Black (13-item) range = 0–13 criteria > 7	# studies (# comparisons)	6 (6)	12 (14)	not applied
		OR (CI)	0.86 (0.59–1.25)	0.56 (0.46 – 0.68)	

OR, odds ratio; CI, 95% confidence interval

The 20 comparisons from the 18 trials included in the head and neck concomitant therapy systematic review yielded 2, 14, 12 and 14 high quality studies, respectively, when the Jadad 3-item, the Jadad 6-item, the Sindhu and the Downs & Black scales was used. Although both Jadad 6-item and Downs & Black scales both assessed 14 comparisons to be from high quality studies, only 11 of these 14 studies were the same. For the 12 comparisons from studies categorized as high quality with the Sindhu scale, 10 of these were also rated high quality by both the Jadad 6-item and the Downs & Black scales, the other 2 were rated as high quality by the Jadad 6-item scale only. There was 1 comparison from a study rated as high quality by the Jadad 6-item scale only and two from studies rated as high quality by the Downs & Black scale only.

#### Impact on pooled estimates of outcome measures

Mortality data (i.e., numbers of deaths and number of patients randomized for each allocation group, abstracted from published trial reports) used for the meta-analysis included in the guideline reports were available for two guidelines and need for blood transfusion data were available for the third [31,32,34]. For each guideline, the pooled odds ratio based on only the high-quality trials was compared with the odds ratio from meta-analysis of all trials that had been included originally in the review (Table 4). For the first guideline [31], there was a significant survival benefit for concomitant chemotherapy and radiotherapy compared with radiotherapy alone for squamous cell head and neck cancer in the meta-analyses that included all studies and the meta-analyses restricted to high quality studies, regardless of quality appraisal tool used. Although the effect size was larger for meta-analysis of high-quality RCTs than for all RCTs (irrespective of quality scale used), the confidence intervals between the two calculations overlapped and the overall conclusions and the recommendations informed by the meta-analysis would have been the same. For the second guideline [32], no survival benefit was detected for adjuvant chemotherapy compared to standard therapy for stage II colon cancer in the meta-analysis of all the studies or the high quality studies, again, regardless of quality appraisal tool used. Although the meta-analysis of the high study quality studies was associated with smaller effect sizes than the calculation including all of the studies, the confidence intervals overlapped and the conclusions and the recommendations would have remained the same.

Only the 6-item Jadad scale was applied to the studies of the EPO guideline and the data were pooled to calculate an overall risk ratio for blood transfusion [34]. The risk ratio for all 15 trials was 0.57 (95% CI, 0.47 to 0.70); for nine trials that scored more than three out of eight on the 6-item Jadad scale, the risk ratio was also 0.57 (95% CI, 0.44 to 0.72) (see Table 4).

## Conclusions

Several conclusions can be drawn from this study and review of the literature. First, there are established methods for assessing the quality of randomized controlled trials in which data on adequate reliability and validity were available. West et al uncovered 32 scales, check lists and component systems concerned with evaluating RCTs [4]; more than the four strategies we applied here. Although most (87%) of the instruments found by West included quality domains for which there is an empirical basis, most failed to report the use rigorous methods in their development and most failed to report data regarding reliability and validity, criteria we set for our study. Interestingly, West et al did not include the Jadad 6-item in their analysis [4], although the Jadad 3-item, Downs & Black, and Sindhu tools were reported.

Although all of the scales we used have established reliability and validity estimates, we found that the number of trials categorized as high quality or low quality depended specifically on the scale that was applied. For the head and neck cancer systematic review, the number comparisons from high quality studies ranged from 2 (when the Jadad 3-item scale was applied) to 14 (when the Jadad 6-item or Downs & Black scales were applied). The range for the colon cancer review was 0 to 9. There was also considerable variability regarding the specific quality category in which each trial was placed. These finding are consistent with those of Juni et al [14] and suggest caution should be applied if the intent of quality rating scales is to restrict the number of studies considered in the systematic review; clearly the choice of scale will have a significant impact regarding what studies are eligible. The problem of identifying to which quality category, high or low, studies should be placed is exacerbated by the lack of clear cut-off criteria identified by the instrument developers. This poses a significant methodological limitation to the utility of these instruments. In our study, we chose the median score as the cut-off criteria in situations where none was reported. However, it would be useful for researchers of these tools to continue the development work to create the evidence-base from which valid criteria can be established.

The lack of consistency of study classification from one scale to the next and the lack of clear cut-off criteria for users to employ when measuring quality of studies, presents a challenge to guideline developers when they need to make choice about which instrument they ought to adopt if the choose to adopt an instrument at all. Rather than clear evidence driving our decisions, we considered other features of the instrument in our decision making. Of the rating scales we examined, our preferred choice would be the Jadad 6-item instrument. In contrast to the others considered in this report, this instrument is relatively easy to implement and interpret and good inter-rater reliability was established. Further, although the 3-item version of the Jadad scale is most commonly used, we found the original 6-item version to be more relevant in our clinical context as it provides greater variation in scores. In the cancer discipline, few trials are placebo-controlled and treatment allocation tends to be poorly reported. In contrast to the pain trials which were profiled in the development phase of the Jadad instrument, the majority of the items in the 3-item version (randomization and blinding items) yield no variation in scores in our context and are, therefore, not useful to discriminate among cancer trials. The 6-item version of the scale more aptly differentiates quality across studies and includes more quality domains for which an empirical basis has been established [4].

Another conclusion that can be drawn from this study is that effect size can be related to study quality but that the nature of the relationship in one clinical area may not generalize to another clinical area. Some of the original work examining the role of study quality reinforces the need to be mindful of the variation among studies included in systematic review [5-7]. However, when we examined five published reviews that had conducted sensitivity analyses on pooled mortality data from RCTs, four of these found that larger effect sizes were associated with high-quality studies, not lower quality trials as has been convention, and the absence or presence of statistical differences between the two allocation groups remained constant. One of challenges in examining this work is that the number of high quality studies is limited; there is a reduction in power that subjects the point estimates to bias. Nonetheless, the potential bias of study design and quality requires thoughtful consideration within a given clinical field.

We conducted sensitivity analysis on the systematic reviews comprising the guidelines developed by the PEBC. Only four systematic reviews among 36 eligible practice guidelines included more than 10 trials with data appropriate for pooling; three from which we could extract data. Although there was some variation in the odds ratios observed, the confidence intervals of the pooled effects from each of the analyses of high quality trials overlapped with the confidence intervals of the pooled odds with all of the trials. In no case would the conclusions based on these results be affected by restricting the meta-analysis to only high quality studies; the recommendations remained the same. Had sensitivity analysis based on study quality been conducted prospectively, it is highly unlikely that different conclusions would have been drawn from the systematic review or that different clinical practice guidelines would have been formulated.

Together, these findings lead to our final conclusion that measuring study quality did not translate into altered conclusions from a systematic review in the oncology domain for the outcomes we used here. Thus, at this time we have decided that measuring study quality using a numerical assessment scale for the purposes of sensitivity analysis will not be a routine part of our guideline development program. We will, however, encourage guideline developers to describe the variation among studies and to point out methodologic flaws. In addition, it will be important for us to repeat this study looking at other outcome measures, such as quality of life and adverse effects, as they become more routinely reported in primary cancer research and incorporated into our practice guidelines. Outcomes other than those studied here may be more sensitive to the issues of study quality.

This study highlights a strategy that may be useful for guideline programs to utilize in making decisions regarding the methods employed in their guideline development process. It is important that scientific inquiry be maintained in studying the value and role of study quality assessment rather than accepting its role as convention. By exploring it within a specific clinical context one can identify it's most appropriate application.

## Competing interests

The author(s) declare that they have no competing interests.

## Authors' contributions

MB, MJ and GB conceived of the project idea and developed the protocol. MB, MJ, and MC conducted by study. SH provided statistical advice and AJ provided conceptual advice. The manuscript was drafted initially by MB and MJ. All authors contributed to the final version submitted for publication.

## Pre-publication history

The pre-publication history for this paper can be accessed here:



## References

[B1] Lohr KN, Carey TS (1999). Assessing "best evidence": issues in grading the quality of studies for systematic reviews. Jt Comm J Qual Improv.

[B2] Clarke M, Oxman AD, editors (2001). Quality assessment of studies. Cochrane Reviewers Handbook 4.1.2 [updated March 2001]; Section 6. The Cochrane Library.

[B3] Moher D, Cook DJ, Jadad AR, Tugwell P, Moher M, Jones A, Pham B, Klassen TP (1999). Assessing the quality of reports of randomised trials: implications for the conduct of meta-analyses. Health Technol Assess.

[B4] West S, King V, Carey TS, Lohr KN, McKoy N, Sutton SF, Lux L (2002). Systems to Rate the Strength of Scientific Evidence. Evidence Report/Technology Assessment No 47 (Prepared by the Research Trial Institute – University of North Carolina Evidence-based Practice Center under Contrast No 290-97-0011) AHRQ Publication No 02-E016.

[B5] Moher D, Pham B, Jones A, Cook DJ, Jadad AR, Moher M, Tugwell P, Klassen TP (1998). Does quality of reports of randomised trials affect estimates of intervention efficacy reported in meta-analyses?. Lancet.

[B6] Schulz KF, Chalmers I, Hayes RJ, Altman DG (1995). Empirical evidence of bias. Dimensions of methodological quality associated with estimates of treatment effects in controlled trials. JAMA.

[B7] Colditz GA, Miller JN, Mosteller F (1989). How study design affects outcomes in comparisons of therapy. Stat Med.

[B8] Ioannidis JP, Lau J (1998). Can quality of clinical trials and meta-analyses be quantified?. Lancet.

[B9] Greenland S (1994). Quality scores are useless and potentially misleading. Am J Epidemiol.

[B10] Emerson JD, Burdick E, Hoaglin DC, Mosteller F, Chalmers TC (1990). An empirical study of the possible relation of treatment differences to quality scores in controlled randomized clinical trials. Control Clin Trials.

[B11] Verhagen AP, de Vet HC, Vermeer F, Widdershoven JWMG, de Bie RA, Kessels AGH, Boers M, van den Brandt PA (2002). The influence of methodologic quality on the conclusion of a landmark meta-analysis on thrombolytic therapy. Int J Technol Assess Health Care.

[B12] Balk EM, Bonis PAL, Moskowitz H, Schmid CH, Ioannidis JPA, Wang C, Lau J (2002). Correlation of quality measures with estimates of treatment effect in meta-analyses of randomized controlled trials. JAMA.

[B13] Verhagen AP, de Vet HC, de Bie RA, Lenssen AF, Kessels AG, Boers M, van den Brandt P (1998). Impact of quality items on study outcome: treatments in acute lateral ankle sprains. Conference Proceedings of the First Symposium on Systematic Reviews: Beyond the Basics.

[B14] Juni P, Witschi A, Bloch R, Egger M (1999). The hazards of scoring the quality of clinical trials for meta-analysis. JAMA.

[B15] Browman GP, Levine MN, Mohide EA, Hayward RS, Pritchard KI, Gafni A, Laupacis A (1995). The practice guidelines development cycle: a conceptual tool for practice guidelines development and implementation. J Clin Oncol.

[B16] Browman GP, Newman TE, Mohide EA, Graham I, Levine MN, Cowan DH (1998). Progress of Clinical Oncology Guidelines Development Using the Practice Guidelines Development Cycle: The Role of Practitioner Feedback. J Clin Oncol.

[B17] Browman G, Brouwers M, De Vito C, Johnston M, Graham I (2000). Participation Patterns of Oncologists in the Development of Clinical Practice Guidelines. Curr Oncol.

[B18] Pater JL, Browman GP, Brouwers MC, Nefsky MF, Evans WK, Cowan DH (2001). Funding New Cancer Drugs in Ontario: Closing the loop in the Practice Guidelines Development Cycle. J Clin Oncol.

[B19] Browman GP (2001). Development and aftercare of clinical guidelines: the balance between rigor and pragmatism. JAMA.

[B20] Moher D, Jadad AR, Tugwell P (1996). Assessing the quality of randomized controlled trials. Current issues and future directions. Int J Technol Assess Health Care.

[B21] Moher D, Jadad AR, Nichol G, Penman M, Tugwell P, Walsh S (1995). Assessing the quality of randomized controlled trials: an annotated bibliography of scales and checklists. Control Clin Trials.

[B22] Jadad AR, Moore RA, Carroll D, Jenkinson C, Reynolds DJ, Gavaghan DJ, McQuay HJ (1996). Assessing the quality of reports of randomized clinical trials: is blinding necessary?. Control Clin Trials.

[B23] Cho MK, Bero LA (1994). Instruments for assessing the quality of drug studies published in the medical literature. JAMA.

[B24] Sindhu F, Carpenter L, Seers K (1997). Development of a tool to rate the quality assessment of randomized controlled trials using a Delphi technique. J Adv Nurs.

[B25] Downs SH, Black N (1998). The feasibility of creating a checklist for the assessment of the methodological quality both of randomised and non-randomised studies of health care interventions. J Epidemiol Community Health.

[B26] McAlister FA, Clark HD, Wells PS, Laupacis A (1998). Perioperative allogeneic blood transfusion does not cause adverse sequelae in patients with cancer: a meta-analysis of unconfounded studies. Br J Surg.

[B27] Caubet JF, Tosteson TD, Dong EW, Naylon EM, Whiting GW, Ernstoff MS, Ross SD (1997). Maximum androgen blockade in advanced prostate cancer: a meta-analysis of published randomized controlled trials using nonsteroidal antiandrogens. Urology.

[B28] Dube S, Heyen F, Jenicek M (1997). Adjuvant chemotherapy in colorectal carcinoma: results of a meta-analysis. Dis Colon Rectum.

[B29] Detsky AS, Naylor CD, O'Rourke K, McGeer AJ, L'Abbe KA (1992). Incorporating variations in the quality of individual randomized trials into meta-analysis. J Clin Epidemiol.

[B30] Klein S, Simes J, Blackburn GL (1986). Total parenteral nutrition and cancer clinical trials. Cancer.

[B31] Browman GP, Hodson DI, Mackenzie RJ, Bestic N, Zuraw L (2001). Choosing a concomitant chemotherapy and radiotherapy regimen for squamous cell head and neck cancer: A systematic review of the published literature with subgroup analysis. Head Neck.

[B32] Figueredo A, Germond C, Maroun J, Browman G, Walker-Dilks C, Wong S, the Gastrointestinal Cancer Disease Site Group (1997). Adjuvant therapy for stage II colon cancer following complete resection. Cancer Prev Control.

[B33] Browman GP, Hodson DI, Newman T, the Head and Neck Cancer Disease Site Group Neoadjuvant chemotherapy in locally advanced squamous cell carcinoma of the head and neck (excluding nasopharynx). Cancer Care Ontario Practice Guidelines Initiative Web site.

[B34] Quirt I, Micucci S, Moran LA, Pater J, Browman G, the Systemic Treatment Disease Site Group (1997). Erythropoietin in the management of cancer patients with non-hematologic malignancies receiving chemotherapy. Cancer Prev Control.

